# Tetra­amminepalladium(II) dichloride ammonia tetra­solvate

**DOI:** 10.1107/S1600536814012355

**Published:** 2014-06-07

**Authors:** Tobias Grassl, Nikolaus Korber

**Affiliations:** a Institut für Anorganische Chemie, Universität Regensburg, Universitätsstrasse 31, 93053 Regensburg, Germany

## Abstract

The title compound, [Pd(NH_3_)_4_]Cl_2_·4NH_3_, was crystallized in liquid ammonia from the salt Pd(en)Cl_2_ (en is ethylenediamine) and is isotypic with [Pt(NH_3_)_4_]Cl_2_·4NH_3_ [Grassl & Korber (2014 ▶). *Acta Cryst.* E**70**, i31]. The Pd^2+^ cation is coordinated by four ammonia mol­ecules, exhibiting a square-planar geometry. The chloride anions are surrounded by nine ammonia mol­ecules. These are either bound in the palladium complex or solvent mol­ecules. The packing of the ammonia solvent mol­ecules enables the formation of an extended network of N—H⋯N and N—H⋯Cl inter­actions with nearly ideal hydrogen-bonding geometry.

## Related literature   

For weak inter­molecular inter­actions such as hydrogen bonds and their application in crystal engeneering, see: Desiraju (2002 ▶); Desiraju (2007 ▶); Steiner (2002 ▶). For the structure of tetra­amminepalladium(II) chloride monoydrate and complexation of palladium by carbohydrates, see: Bell *et al.* (1976 ▶); Ahlrichs *et al.* (1998 ▶). The structure of the platinum analogue is given by Grassl & Korber (2014 ▶)

## Experimental   

### 

#### Crystal data  

[Pd(NH_3_)_4_]Cl_2_·4NH_3_
*M* *_r_* = 313.58
Monoclinic, 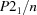

*a* = 7.6856 (5) Å
*b* = 10.1505 (7) Å
*c* = 8.7170 (6) Å
β = 100.384 (7)°
*V* = 668.90 (8) Å^3^
*Z* = 2
Mo *K*α radiation
μ = 1.76 mm^−1^
*T* = 123 K
0.32 × 0.29 × 0.23 mm

#### Data collection  

Agilent Xcalibur (Ruby, Gemini ultra) diffractometer
Absorption correction: analytical [*CrysAlis PRO* (Agilent, 2012 ▶), using a multi-faceted crystal model based on expressions derived by Clark & Reid (1995 ▶)] *T*_min_ = 0.649, *T*_max_ = 0.741
2418 measured reflections
1266 independent reflections
1076 reflections with *I* > 2σ(*I*)
*R*_int_ = 0.034

#### Refinement  

*R*[*F* ^2^ > 2σ(*F* ^2^)] = 0.027
*wR*(*F* ^2^) = 0.058
*S* = 1.06
1266 reflections
100 parameters
All H-atom parameters refined
Δρ_max_ = 0.45 e Å^−3^
Δρ_min_ = −0.55 e Å^−3^

### 

Data collection: *CrysAlis PRO* (Agilent, 2012 ▶); cell refinement: *CrysAlis PRO*; data reduction: *CrysAlis PRO*; program(s) used to solve structure: *OLEX2.solve* (Bourhis *et al.*, 2014 ▶); program(s) used to refine structure: *SHELXL97* (Sheldrick, 2008 ▶); molecular graphics: *DIAMOND* (Brandenburg & Putz, 2012 ▶); software used to prepare material for publication: *OLEX2* (Dolomanov *et al.*, 2009 ▶).

## Supplementary Material

Crystal structure: contains datablock(s) I. DOI: 10.1107/S1600536814012355/pk2523sup1.cif

Structure factors: contains datablock(s) I. DOI: 10.1107/S1600536814012355/pk2523Isup2.hkl

Supporting information file. DOI: 10.1107/S1600536814012355/pk2523Isup3.mol

CCDC reference: 1005539

Additional supporting information:  crystallographic information; 3D view; checkCIF report

## Figures and Tables

**Table 1:** Hydrogen-bond geometry (Å, °)

*D*—H⋯*A*	*D*—H	H⋯*A*	*D*⋯*A*	*D*—H⋯*A*
N1—H1*A*⋯Cl1	0.86 (4)	2.49 (4)	3.351 (3)	175 (3)
N1—H1*B*⋯Cl1^i^	0.85 (3)	2.49 (4)	3.328 (3)	171 (3)
N2—H2*A*⋯N3^ii^	1.03 (4)	2.02 (4)	3.025 (5)	163 (3)
N1—H1*C*⋯N4	0.96 (4)	2.02 (5)	2.975 (5)	170 (3)
N2—H2*B*⋯Cl1^i^	0.73 (3)	2.66 (3)	3.384 (4)	172 (3)
N2—H2*C*⋯Cl1^iii^	0.88 (5)	2.62 (5)	3.463 (3)	162 (4)
N3—H3*A*⋯Cl1	0.83 (3)	2.83 (3)	3.563 (4)	148 (3)
N4—H4*A*⋯Cl1^iv^	0.91 (4)	2.65 (4)	3.563 (4)	173 (3)
N4—H4*B*⋯Cl1^v^	1.00 (5)	2.61 (5)	3.606 (4)	174 (4)
N3—H3*B*⋯Cl1^vi^	0.89 (5)	2.71 (5)	3.578 (4)	163 (3)
N3—H3*C*⋯Cl1^vii^	1.09 (5)	2.48 (5)	3.535 (4)	162 (4)

Symmetry codes: (i) 
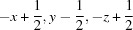
; (ii) 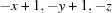; (iii) 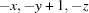; (iv) 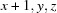; (v) 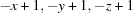; (vi) 
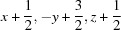
; (vii) 
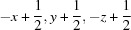
.

**Fig. 1.**
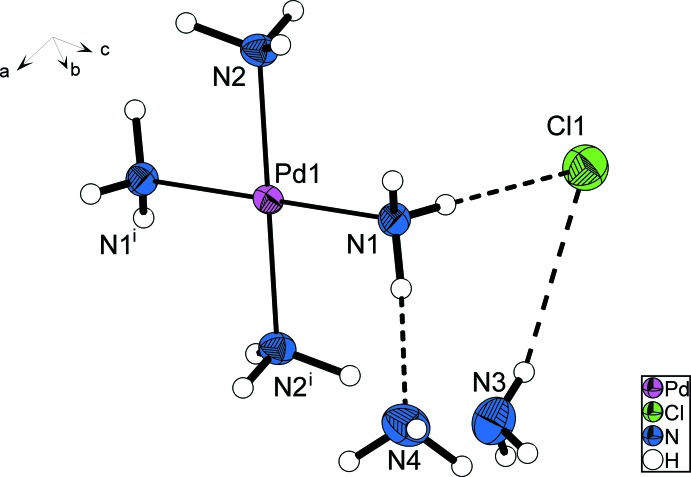
: Crystal structure of the title compound with labeling and displacement ellipsoids drawn at the 50% probability level. Symmetry code: (i) 1 - x, 1 - y, - z.

**Fig. 2.**
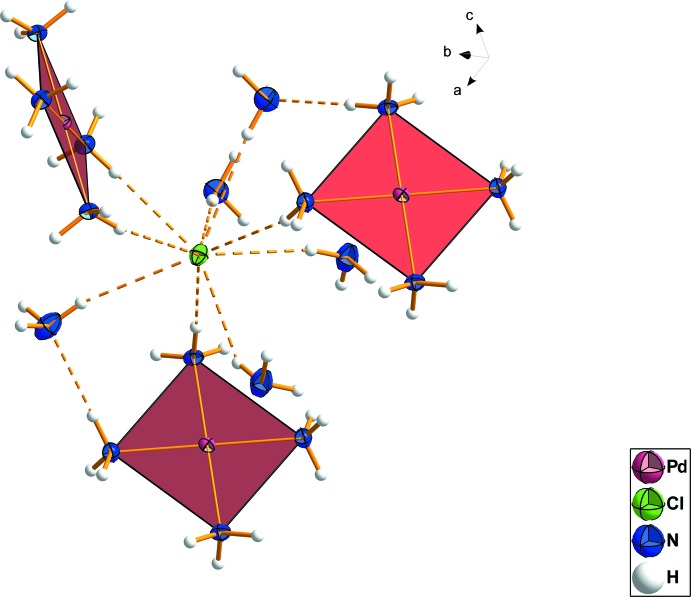
: The chloride anion is shown with its surrounding molecules. The predominant bond type is hydrogen bonding. Displacement ellipsoids are drawn at the 50% probability level.

**Fig. 3.**
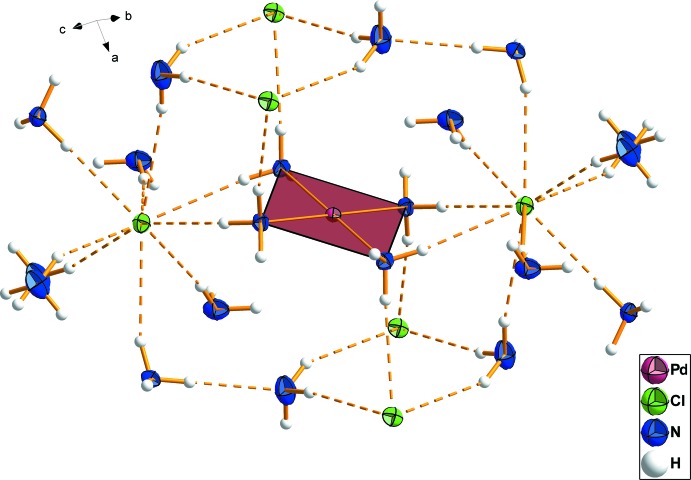
: Extended network of hydrogen bonds in the crystal structure. The solvent ammonia molecules are oriented to optimize the hydrogen bond geometry. Displacement ellipsoids are drawn at the 50% probability level.

## supplementary crystallographic information

### S1. Comment

The crystal structure of the title compound was determined in the course of investigations into the reactivity of carbohydrates towards metal cations in liquid ammonia.

As in the platinum compound, the palladium cation forms a homoleptic ammine complex with a square-planar coordination geometry. Pd—N bond lengths are 2.032 (3) Å and 2.048 (3) Å, respectively, while the angles N—Pd—N are 88.59 (13)° and 91.41 (13)°. Ammonia ligands opposite to each other within the complex cation have staggered hydrogen atom positions (Fig. 1).

The chloride anion exhibits nine contacts to hydrogen atoms of ammonia molecules which are either bound in the complex or solvate molecules, forming a network of hydrogen bonds (Fig. 2 and Fig. 3). Bond angles (N—H···Cl) are between 148 (3)° and 175 (3)° whereas N—H···Cl bond lengths are observed with values between 2.48 (5) Å and 2.83 (3) Å. The two N—H···N bridges are close to 180°, with bond angles of 163 (3)° and 170 (3)° and bond lengths significantly less than the sum of the van der Waals radii of nitrogen and hydrogen (2.02 (4) Å and 2.02 (5) Å). These observations give strong evidence that a significant energy contribution from the hydrogen bond network drives the arrangement of the overall structure.

### S2. Experimental

0.25 g (1.05 mmol) Pd(en)Cl_2_ and 0.188 g (1.05 mmol) *D*-(+)-glucono-1,5-lactone were placed under argon atmosphere in a reaction flask and 50 ml of dry liquid ammonia were condensed. This mixture was stored in a refrigerator at 237 K for one week to ensure that all substances were completely dissolved. The flask was then stored at 161 K for five months. After that period of time, clear colorless crystals of the title compound were found on the wall of the reaction vessel.

### S3. Refinement

The crystal structure does not show any features where special refinement procedures had to be applied. All hydrogen atoms were located in difference maps and both bond angle/bond length and isotropic displacement parameters were refined.

### Crystal data

**Table:**

[Pd(NH_3_)_4_]Cl_2_·4NH_3_	*F*(000) = 320
*M**_r_* = 313.58	*D*_x_ = 1.557 Mg m^−^^3^
Monoclinic, *P*2_1_/*n*	Mo *K*α radiation, λ = 0.71073 Å
*a* = 7.6856 (5) Å	Cell parameters from 1429 reflections
*b* = 10.1505 (7) Å	θ = 3.1–29.3°
*c* = 8.7170 (6) Å	µ = 1.76 mm^−^^1^
β = 100.384 (7)°	*T* = 123 K
*V* = 668.90 (8) Å^3^	Block, clear colourless
*Z* = 2	0.32 × 0.29 × 0.23 mm

### Data collection

**Table:**

Agilent Xcalibur (Ruby, Gemini ultra) diffractometer	1266 independent reflections
Radiation source: fine-focus sealed tube	1076 reflections with *I* > 2σ(*I*)
Graphite monochromator	*R*_int_ = 0.034
phi and ω scans	θ_max_ = 25.7°, θ_min_ = 3.1°
Absorption correction: analytical [*CrysAlis PRO* (Agilent, 2012), using a multi-faceted crystal model based on expressions derived by Clark & Reid (1995)]	*h* = −7→9
*T*_min_ = 0.649, *T*_max_ = 0.741	*k* = −12→12
2418 measured reflections	*l* = −10→10

### Refinement

**Table:**

Refinement on *F*^2^	Primary atom site location: iterative
Least-squares matrix: full	Secondary atom site location: difference Fourier map
*R*[*F*^2^ > 2σ(*F*^2^)] = 0.027	Hydrogen site location: inferred from neighbouring sites
*wR*(*F*^2^) = 0.058	All H-atom parameters refined
*S* = 1.06	*w* = 1/[σ^2^(*F*_o_^2^) + (0.0075*P*)^2^] where *P* = (*F*_o_^2^ + 2*F*_c_^2^)/3
1266 reflections	(Δ/σ)_max_ < 0.001
100 parameters	Δρ_max_ = 0.45 e Å^−^^3^
0 restraints	Δρ_min_ = −0.55 e Å^−^^3^

### Special details

**Table:**

Experimental. Absorption correction: CrysAlisPro, Agilent Technologies, Version 1.171.35.21 Analytical numeric absorption correction using a multifaceted crystal model based on expressions derived by R.C. Clark & J.S. Reid. (Clark & Reid, 1995)
Geometry. All e.s.d.'s (except the e.s.d. in the dihedral angle between two l.s. planes) are estimated using the full covariance matrix. The cell e.s.d.'s are taken into account individually in the estimation of e.s.d.'s in distances, angles and torsion angles; correlations between e.s.d.'s in cell parameters are only used when they are defined by crystal symmetry. An approximate (isotropic) treatment of cell e.s.d.'s is used for estimating e.s.d.'s involving l.s. planes.
Refinement. Refinement of *F*^2^ against ALL reflections. The weighted *R*-factor *wR* and goodness of fit *S* are based on *F*^2^, conventional *R*-factors *R* are based on *F*, with *F* set to zero for negative *F*^2^. The threshold expression of *F*^2^ > σ(*F*^2^) is used only for calculating *R*-factors(gt) *etc*. and is not relevant to the choice of reflections for refinement. *R*-factors based on *F*^2^ are statistically about twice as large as those based on *F*, and *R*- factors based on ALL data will be even larger.

### Fractional atomic coordinates and isotropic or equivalent isotropic displacement parameters (Å^2^)

**Table:**

	*x*	*y*	*z*	*U*_iso_*/*U*_eq_	
Pd1	0.5000	0.5000	0.0000	0.01592 (13)	
Cl1	0.09460 (10)	0.66086 (7)	0.23005 (10)	0.0236 (2)	
N1	0.4602 (4)	0.4827 (3)	0.2233 (3)	0.0200 (6)	
N2	0.3507 (4)	0.3356 (3)	−0.0659 (4)	0.0219 (6)	
N3	0.5125 (5)	0.8226 (3)	0.3132 (4)	0.0358 (8)	
N4	0.7709 (4)	0.5695 (4)	0.4561 (4)	0.0353 (8)	
H1A	0.364 (5)	0.525 (3)	0.229 (5)	0.038 (12)*	
H1B	0.449 (4)	0.402 (3)	0.246 (4)	0.024 (10)*	
H2A	0.382 (4)	0.294 (3)	−0.166 (5)	0.036 (10)*	
H1C	0.556 (6)	0.521 (3)	0.296 (5)	0.046 (12)*	
H2B	0.358 (4)	0.292 (3)	0.001 (4)	0.018 (11)*	
H2C	0.238 (6)	0.355 (4)	−0.097 (5)	0.064 (14)*	
H3A	0.403 (5)	0.818 (3)	0.287 (4)	0.017 (9)*	
H4A	0.861 (5)	0.594 (3)	0.406 (4)	0.028 (10)*	
H4B	0.817 (6)	0.507 (4)	0.541 (6)	0.066 (15)*	
H3B	0.554 (6)	0.815 (4)	0.415 (6)	0.055 (14)*	
H4C	0.751 (5)	0.646 (4)	0.517 (5)	0.062 (14)*	
H3C	0.493 (6)	0.925 (5)	0.277 (6)	0.086 (16)*	

### Atomic displacement parameters (Å^2^)

**Table:**

	*U*^11^	*U*^22^	*U*^33^	*U*^12^	*U*^13^	*U*^23^
Pd1	0.01442 (19)	0.0156 (2)	0.0177 (2)	−0.00015 (13)	0.00265 (14)	0.00067 (14)
Cl1	0.0235 (4)	0.0204 (4)	0.0260 (4)	0.0014 (4)	0.0024 (3)	−0.0023 (4)
N1	0.0242 (16)	0.0182 (16)	0.0186 (16)	−0.0026 (14)	0.0069 (13)	0.0011 (13)
N2	0.0226 (16)	0.0219 (16)	0.0210 (17)	−0.0041 (14)	0.0031 (14)	0.0023 (15)
N3	0.041 (2)	0.035 (2)	0.031 (2)	0.0050 (17)	0.0056 (17)	0.0004 (17)
N4	0.0296 (17)	0.042 (2)	0.0320 (19)	−0.0026 (17)	−0.0002 (15)	0.0059 (18)

### Geometric parameters (Å, º)

**Table:**

Pd1—N1^i^	2.032 (3)	N2—H2B	0.73 (3)
Pd1—N1	2.032 (3)	N2—H2C	0.88 (5)
Pd1—N2^i^	2.048 (3)	N3—H3A	0.83 (3)
Pd1—N2	2.048 (3)	N3—H3B	0.89 (5)
N1—H1A	0.86 (4)	N3—H3C	1.09 (5)
N1—H1B	0.85 (3)	N4—H4A	0.91 (4)
N1—H1C	0.96 (4)	N4—H4B	1.00 (5)
N2—H2A	1.03 (4)	N4—H4C	0.97 (4)
			
N1—Pd1—N1^i^	179.999 (1)	Pd1—N2—H2A	111.2 (18)
N1—Pd1—N2^i^	88.59 (13)	Pd1—N2—H2B	109 (3)
N1^i^—Pd1—N2^i^	91.41 (13)	Pd1—N2—H2C	112 (3)
N1^i^—Pd1—N2	88.59 (13)	H2A—N2—H2B	115 (3)
N1—Pd1—N2	91.41 (13)	H2A—N2—H2C	102 (3)
N2—Pd1—N2^i^	180.00 (10)	H2B—N2—H2C	108 (4)
Pd1—N1—H1A	107 (3)	H3A—N3—H3B	115 (4)
Pd1—N1—H1B	110 (2)	H3A—N3—H3C	84 (3)
Pd1—N1—H1C	112 (3)	H3B—N3—H3C	112 (4)
H1A—N1—H1B	110 (3)	H4A—N4—H4B	109 (3)
H1A—N1—H1C	108 (3)	H4A—N4—H4C	105 (3)
H1B—N1—H1C	109 (3)	H4B—N4—H4C	100 (4)

Symmetry code: (i) −*x*+1, −*y*+1, −*z*.

### Hydrogen-bond geometry (Å, º)

**Table:**

*D*—H···*A*	*D*—H	H···*A*	*D*···*A*	*D*—H···*A*
N1—H1*A*···Cl1	0.86 (4)	2.49 (4)	3.351 (3)	175 (3)
N1—H1*B*···Cl1^ii^	0.85 (3)	2.49 (4)	3.328 (3)	171 (3)
N2—H2*A*···N3^i^	1.03 (4)	2.02 (4)	3.025 (5)	163 (3)
N1—H1*C*···N4	0.96 (4)	2.02 (5)	2.975 (5)	170 (3)
N2—H2*B*···Cl1^ii^	0.73 (3)	2.66 (3)	3.384 (4)	172 (3)
N2—H2*C*···Cl1^iii^	0.88 (5)	2.62 (5)	3.463 (3)	162 (4)
N3—H3*A*···Cl1	0.83 (3)	2.83 (3)	3.563 (4)	148 (3)
N4—H4*A*···Cl1^iv^	0.91 (4)	2.65 (4)	3.563 (4)	173 (3)
N4—H4*B*···Cl1^v^	1.00 (5)	2.61 (5)	3.606 (4)	174 (4)
N3—H3*B*···Cl1^vi^	0.89 (5)	2.71 (5)	3.578 (4)	163 (3)
N3—H3*C*···Cl1^vii^	1.09 (5)	2.48 (5)	3.535 (4)	162 (4)

Symmetry codes: (i) −*x*+1, −*y*+1, −*z*; (ii) −*x*+1/2, *y*−1/2, −*z*+1/2; (iii) −*x*, −*y*+1, −*z*; (iv) *x*+1, *y*, *z*; (v) −*x*+1, −*y*+1, −*z*+1; (vi) *x*+1/2, −*y*+3/2, *z*+1/2; (vii) −*x*+1/2, *y*+1/2, −*z*+1/2.
